# Usefulness of Myocardial Injury Parameters in Predicting Prolonged Postoperative Use of Inotropes Drugs in Patients Undergoing Heart Valve Surgery

**DOI:** 10.3390/jcm14082719

**Published:** 2025-04-15

**Authors:** Piotr Duchnowski, Witold Śmigielski, Piotr Kołsut

**Affiliations:** Cardinal Wyszynski National Institute of Cardiology, 04-628 Warsaw, Polandpkolsut@ikard.pl (P.K.)

**Keywords:** heart valve surgery, prolonged postoperative use of inotropes drugs, low cardiac output syndrome, N-terminal of the prohormone brain natriuretic peptide (NT-proBNP), Troponin T

## Abstract

**Background:** Prolonged use of inotropes drugs in the early postoperative period is one of the most common complications occurring in patients undergoing heart valve surgery. Patients requiring prolonged support via inotropes drugs are significantly more likely to experience serious postoperative complications such as acute kidney injury, cardiogenic shock, multiple organ dysfunction syndrome, and death. This study assessed the usefulness of selected perioperative parameters in predicting prolonged postoperative use of inotropic drugs and cardiogenic shock and/or death in a group of patients requiring prolonged supply of inotropes drugs. **Methods:** This prospective study was conducted on a group of 607 patients undergoing heart valve surgery. The primary endpoint in-hospital follow-up was prolonged postoperative use of inotropes drugs. The secondary composite endpoint was cardiogenic shock requiring mechanical circulatory support (MCS) and/or death from any cause in patients with prolonged postoperative use of inotropes drugs. **Results:** A total of 210 patients required inotropes drugs for more than 48 h. Age (*p* = 0.03), preoperative atrial fibrillation (*p* < 0.001), preoperative NT-proBNP level (*p* < 0.001), Troponin T measured one day after surgery (TnT II) (*p* < 0.001), and the need for urgent postoperative rethoracotomy (*p* < 0.001) remained independent predictors of primary endpoint. Preoperative hemoglobin level (*p* = 0.001) and TnT II (*p* < 0.001) were independent predictors of death and cardiogenic shock requiring MCS. **Conclusions:** Patients with elevated preoperative NT-proBNP values, as well as with increasing postoperative troponin T levels, are at risk of prolonged postoperative use of inotropes drugs, a complication which is associated with a significant risk of developing further adverse consequences, such as cardiogenic shock and death.

## 1. Introduction

One of the most common complications occurring in the early postoperative period in patients undergoing heart valve surgery is postoperative prolonged use of inotropes drugs, which most often results from low cardiac output syndrome (LCOS) [1,2,3,4]. The definition of LCOS includes a reduction in cardiac index (CI) to <2.0 L/min/m^2^ and systolic blood pressure <90 mm Hg in the absence of hypovolemia [5,6,7,8,9]. To improve the patient’s hemodynamics, it is usually necessary, in addition to the patient’s fluid balance, to use inotropes drugs and sometimes also mechanical circulatory support (MCS). LCOS is often a critical point in the early postoperative period, as it may lead to the development of further unfavorable consequences, including the development of individual organ failure, including acute kidney injury (AKI) requiring renal replacement therapy, cardiogenic shock requiring mechanical circulatory support (MCS), or multiple organ dysfunction syndrome (MODS) [10,11,12,13]. In addition, LCOS often leads to increased use of hospital resources, extended total hospitalization time and stay in the intensive care unit, and, ultimately, increased mortality. Therefore, rapid identification of LCOS is essential to enable rapid identification of the cause as well as the implementation of immediate goal-directed therapy, which includes, among other measures, improved oxygen delivery to peripheral tissues, leading to improved tissue metabolism and organ function, ultimately improving clinical outcomes. To date, information on predictors of prolonged postoperative use of inotropes drugs is limited in the available literature [14,15,16,17,18]. In recent years, both the diagnostics and prognosis of many cardiovascular diseases have attracted considerable interest in markers of myocardial overload and damage, i.e., high-sensitivity troponin T (hs-TnT) and N-terminal propeptide B-type natriuretic peptide (NT-proBNP), the use of which is now part of the daily practice of every cardiologist [19].

The main aim of this article was to evaluate the predictive abilities of selected perioperative parameters in terms of their ability to predict the occurrence of prolonged postoperative use of inotropes drugs in patients undergoing heart valve surgery.

## 2. Methods

This was a prospective study conducted on a group of patients with hemodynamically severe valvular heart disease treated via cardiac surgery at the Cardinal Wyszynski National Institute of Cardiology in Warsaw, Poland, in 2014–2021. The exclusion criteria for participation in the study were as follows: being below 18 years of age, the lack of consent to participate in the study, the presence of a porcelain aorta, significantly atherosclerotic changes in the carotid arteries described in the imaging study, the presence of autoimmune diseases, the presence of chronic intestinal inflammation, and the presence of active disease cancer. A blood sample was collected from each patient before the procedure, immediately after the patient arrived at the intensive care unit after the procedure, and on the morning of the first day after the procedure to determine blood count parameters and biochemical tests. Test electrochemiluminescent immunoassays (Elecsys 2010 (Roche, Munich, Germany)) were used to determine the level of the N-terminal segment of the prohormone of brain natriuretic peptide (NT-proBNP). In the center conducting the study, the normal level of NT-proBNP is <125 pg/mL. In turn, the Troponin hs-STAT test (Roche) was used to determine three high-sensitivity plasma troponin T measurements: TnT—troponin T measured before surgery; TnT I—troponin T measured immediately after surgery; and TnT II—troponin T measured one day after surgery. The procedure was performed under general anesthesia using extracorporeal circulation. The primary endpoint of in-hospital follow-up was prolonged postoperative use of inotropes drugs (such as dobutamine, epinephrine, norepinephrine, dopamine, levosimendan, and/or milrinone) defined as the need to take inotropes drugs for more than 48 h due to persistent systolic blood pressure of <90 mmHg combined with evidence of tissue hypoperfusion in the absence of hypovolemia. The secondary composite endpoint consisted of cardiogenic shock requiring mechanical circulatory support and/or death from any cause from among patients with prolonged postoperative use of inotropes drugs. The observation period of patients included in the study was until discharge from the hospital or until death occurred during the current hospitalization. The Bioethics Committee at the Cardinal Wyszynski National Institute of Cardiology in Warsaw, Poland gave its consent to conduct the study, which was designated the number 2.32/VI/18. Each patient included in the study signed the consent form.

### Statistical Analysis

Median (Q1–Q3) and frequencies (%) were used to represent the collected data. The Mann–Whitney U test for quantitative variables and the chi-square test for qualitative variables were used to assess intergroup cooperation. When the distribution of the analyzed variables differed from the normal distribution, a nonparametric test (the Shapiro–Wilk test) was used. To determine predictors of the primary and secondary endpoint, univariable logistic regression analysis was used. Statistically significant variables obtained in the univariable analysis were used to perform multivariable logistic regression analysis. Receiver operating characteristic (ROC) curve analysis was used to assess the predictive ability of quantitative variables identified in multivariate logical regression analysis for the occurrence of endpoints. Pearson correlation coefficient was used to search for associations between the studied variables. Statistical analyses were performed using the STATISTICA 12 software (StatSoft Polska Sp. z o.o.; Kraków, Poland). The significance level was set as *p* < 0.05.

In our simulations, we defined the event rate as 15%, so assuming the power of the sample (1-β err prob) was 0.95 and α err prob and β err prob were 0.049, the study group of patients should have comprised at least 570 patients.

## 3. Results

**Figure 1:**
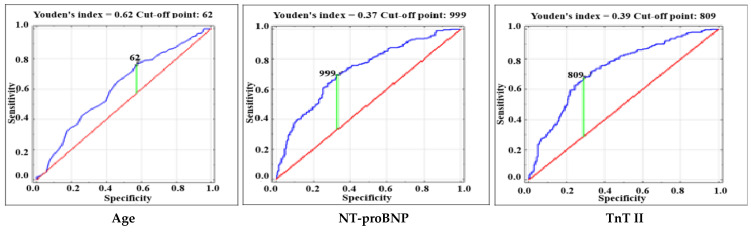
Receiver operating characteristic (ROC) curves for age, preoperative NT-proBNP level and Troponin T measured on the 1st postoperative day (TnT II) for the occurrence of the primary endpoint.

**Figure 2:**
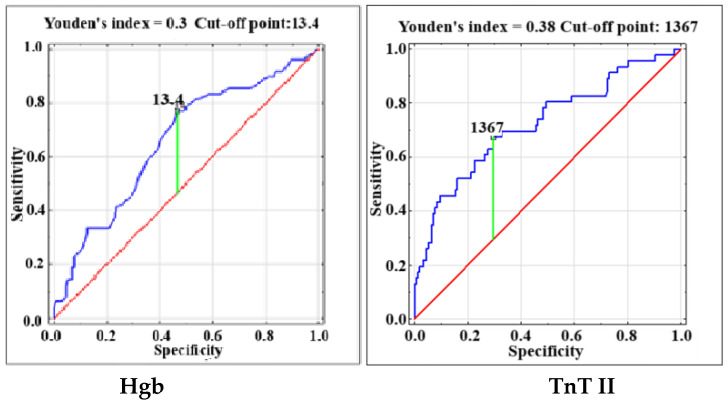
Receiver operating characteristic (ROC) curves for preoperative hemoglobin level (Hgb) and Troponin T measured on the 1st postoperative day (TnT II) for the occurrence of the secondary endpoint.

The present study included 607 patients undergoing heart valve surgery at the National Institute of Cardiology in Warsaw, Poland. The characteristics of the entire group of patients, including the division into subgroups of patients with or without the primary endpoint, are presented in Table 1. The primary endpoint was observed in 210 patients. Table 2 presents statistically significant predictors of the primary endpoint. In the multivariable analysis, age (OR 1.022; 95% CI 1.002–1.042; *p* = 0.03), preoperative atrial fibrillation (OR 3.072; 95% CI 1.982–4.759; *p* < 0.001), preoperative NT-proBNP level (OR 1.570; 95% CI 1.304–1.891; *p* < 0.001), troponin T measured one day after surgery (TnT II) (OR 1.933; 95% CI 1.492–2.506; *p* < 0.001), and the need for urgent postoperative rethoracotomy (OR 5.518; 95% CI 2.800–10.876; *p* < 0.001) remained independent predictors of the primary endpoint. In the group of 210 patients with prolonged postoperative use of inotropes drugs, serious postoperative complications occurred significantly more often. These included the need for emergency rethoracotomy and re-intubation, postoperative stroke, acute kidney injury requiring the use of renal replacement therapy, cardiogenic shock requiring the use of mechanical circulatory support, multiple-organ dysfunction syndrome, as well as 30-day mortality, compared to the group of patients without prolonged postoperative use of catecholamines. Moreover, patients with prolonged postoperative use of inotropes drugs were characterized by a longer hospital stay and higher troponin T levels measured in the following postoperative hours. The median duration of pressor administration was 5 (3–10) days. Statistical analyses showed that none of the procedures listed in Table 1 were, per se, associated with a significantly higher incidence of postoperative hemodynamic instability. The secondary endpoint occurred in 48 patients, including 21 patients with cardiogenic shock requiring mechanical circulatory support, and/or death in 38 patients. Table 3 shows predictors of the secondary endpoint. TnT II (OR 3.413; 95% CI 2.328–5.005; *p* < 0.001) and preoperative hemoglobin level (OR 0.696; 95% CI 0.558–0.867; *p* = 0.001) were independent predictors of death and/or cardiogenic shock requiring MCS. The correlation coefficient between independent predictors of both the primary and secondary endpoints did not exceed 0.11. Figure 1 and Figure 2 present the ROC curves of independent predictors (quantitative variables) of the primary and secondary endpoints.

## 4. Discussion

In this study, as many as 210 patients required prolonged support with inotropes drugs (34% of the entire study group). It is worth noting that in the group of patients with prolonged postoperative use of inotropes drugs, serious postoperative complications were significantly more frequently observed. These included postoperative stroke, acute kidney injury requiring renal replacement therapy, full-blown cardiogenic shock, or multi-organ failure syndrome. In addition, the occurrence of the primary endpoint was associated with a significantly longer hospital stay as well as increased 30-day mortality. Due to the lack of stabilization of the circulatory system, 21 patients developed cardiogenic shock requiring the use of mechanical circulatory support, while 38 patients died, mainly due to the development of multi-organ dysfunction syndrome (MODS). The results of the statistical analyses performed indicated that the independent predictors of prolonged postoperative use of inotropes drugs were the patient’s age, the presence of atrial fibrillation in the preoperative period, the preoperative NT-proBNP level, the need to perform an urgent postoperative rethoracotomy, and an increasing level of postoperative troponin T. In addition, the risk factors for postoperative hemodynamic instability were higher EuroSCORE II calculation score, female gender, lower preoperative hemoglobin level and GFR, as well as higher preoperative troponin T and CRP levels, moreover longer duration of extracorporeal circulation during surgery. In turn, the preoperative hemoglobin level and elevated TnT II level were independent predictors of death and cardiogenic shock.

Cardiac output, i.e., the amount of blood pumped by the left ventricle in 1 min, and blood morphology parameters are the main determinants of the ability to deliver oxygen to all body cells [11,17]. According to the available literature and the results of the above study, postoperative hemodynamic instability is one of the most common complications observed in the early postoperative period in patients undergoing heart valve surgery [10,13,20]. Insufficient cardiac output causes the function of the cardiovascular system to become unreliable and insufficient, which may result in a reduction in oxygen supply in peripheral tissues, including important internal organs, which may lead to deterioration in cell metabolism, cell damage and, subsequently, deterioration of their function, which translates into the occurrence of symptoms of acute kidney injury, increasing liver parameters, central nervous system hypoxia, development of respiratory failure, impaired hematopoiesis and hemostasis, cardiogenic shock, which, like a domino effect, may result in the development of multi-organ failure syndrome—an extremely serious clinical condition, often associated with irreversible tissue damage leading to death [11]. It is worth noting that the state of hemodynamic instability itself forces the patient to be immobilized most often in the intensive care unit, which contributes to impaired lung aeration, an increased risk of infection with multi-drug-resistant microorganisms, muscle weakness and thromboembolic complications, etc. [21,22].

So far, there is little information in the available literature on the predictive factors of postoperative hemodynamic instability in patients undergoing cardiac surgery due to valvular heart disease [1,4,5,13]. The obtained results indicate that the overloaded myocardium in a patient with severe valvular heart disease, expressed by elevated preoperative NT-proBNP values as well as the presence of atrial fibrillation, is particularly sensitive to non-physiological perioperative conditions related to the use of extracorporeal circulation, cardioplegia or blood loss in the perioperative period, which may ultimately lead to cardiovascular failure in the early postoperative period. On the other hand, increasing postoperative Troponin T levels, indicating intraoperative myocardial damage, are also associated with an increased risk of postoperative hemodynamic instability and the possibility of developing further unfavorable consequences [13,23,24,25,26]. It is worth noting that a significant increase in TnT I and TnT II levels may be associated with the development of periprocedural myocardial infarction, which is quite common and may contribute to the development of postoperative hemodynamic instability [27].

The results of the presented study therefore indicate that the need for prolonged use of inotropes drugs in the early postoperative period is a condition that determines further possible events; therefore, a meticulous approach and the implementation of quick, accurate diagnostic and therapeutic solutions may influence the patient’s fate. Patients with higher preoperative NT-proBNP levels as well as increasing Troponin T levels in the postoperative period, additional burdens such as advanced age, the presence of atrial fibrillation, as well as a higher risk of surgery estimated using the EuroSCORE II model should be given special attention in the perioperative period, as early diagnosis of developing postoperative hemodynamic instability gives the patient a chance to immediately implement adequate therapy, which may improve tissue perfusion and, thus, the function of individual organs and systems, as well as stopping the unfavorable cascade, which may ultimately improve treatment results.

The presented study has several limitations. It is a single-center study. The study included a limited number of patients because the principal investigator personally recruited patients and was unable to include all consecutive patients undergoing cardiac surgery at the center during the given time period, and therefore the study may be limited by selection bias as well as any differences in the center where the study was conducted, which may affect the overall picture of treatment. Due to the limited number of patients included in the study, analyses were not performed for individual valvular heart defects, and the analyses did not include complications such as periprocedural myocardial infarction, sepsis, or brady- and tachyarrhythmias. It should be taken into account that factors such as periprocedural myocardial infarction, postoperative sepsis, and bradyarrhythmias, which are commonly observed in patients after heart valve surgery, may be the cause of postoperative hemodynamic instability requiring the administration of inotropes drugs and may also be the cause of increased parameters of postoperative myocardial damage. Therefore, in future studies, increasing the number of patients recruited to the study as well as expanding the number of centers participating in the study may enable confirmation of the obtained results.

## 5. Conclusions

This study showed that myocardial injury parameters such as elevated preoperative NT-proBNP levels as well as increasing postoperative troponin T levels may be useful for predicting postoperative hemodynamic instability requiring prolonged inotropic support in the early postoperative period in patients undergoing heart valve surgery. In turn, elevated postoperative troponin T levels as well as lower preoperative hemoglobin values indicate a higher risk of cardiogenic shock and death in patients with postoperative hemodynamic instability requiring prolonged inotropic support. The study has some limitations. It was a single-center study with a limited number of patients included in the study. In the future, increasing the number of centers and the group of patients studied may confirm the obtained results.

## Figures and Tables

**Table 1:** Baseline characteristics of the study population (n = 607).

Preoperative Characteristics of Patients	Values All Patients	Values Patients with Hemodynamic Instability (*n* = 210)	Values Patients Without Hemodynamic Instability (*n* = 397)	*p*-Value
Age, years	65 (57–71)	67 (62–74)	63 (55–70)	0.01
Male: men, *n* (%)	352 (58)	108 (52)	244 (62)	0.02
LV ejection fraction, (%)	60 (50–65)	55 (45–65)	60 (55–65)	<0.001
EuroSCORE II, %	2.4 (1.4–3.9)	3.4 (1.7–5.1)	2.0 (1.0–3.0)	<0.001
Atrial fibrillation, *n* (%)	237 (39)	136 (64)	101 (25)	<0.001
Previous myocardial infarction, *n* (%)	39 (6)	22 (10)	17 (4)	0.02
Diabetes mellitus, *n* (%)	97 (15)	42 (20)	55 (13)	0.08
Hemoglobin, g/dL	13.7 (12.7–14.6)	13.3 (12.1–14.5)	13.9 (12.9–14.9)	<0.001
GFR, mmol/L	66.8 (55–81)	60 (47.5–72.5)	70 (56.5–83.5)	<0.001
TnT, ng/L	12.4 (7–30)	18.5 (7–30)	11.1(4.6–17.6)	<0.001
NT-proBNP, pg/mL	895 (295–1945)	1618 (757–3217)	602 (230–1432)	<0.001
CRP, mg/dL	0.2 (0.1–0.5)	0.3 (0.2–0.7)	0.2 (0.1–0.3)	<0.001
Aortic cross-clamp time, min	110 (63–130)	122 (84–160)	80 (55–105)	0.06
Cardiopulmonary bypass time, min	115 (82–130)	168 (129–208)	103 (79–127)	0.007
Postoperative characteristics of patients				
Rethoracotomy, *n* (%)	78 (13)	56 (26)	22 (6)	<0.001
Postoperative stroke, *n* (%)	21 (3.4)	18 (8)	3 (1)	<0.001
Re-intubation, *n* (%)	83 (13)	71 (33)	12 (3)	<0.001
Renal replacement therapy, *n* (%)	43 (7)	42 (20)	1 (0.2)	<0.001
Mechanical circulatory support, *n* (%)	21 (3.4)	21 (10)	0 (0)	<0.001
Multiple-organ dysfunction syndrome, *n* (%)	40 (6)	37 (17)	3 (1)	<0.001
Hospital stay after surgery, day	11 (8–17)	16 (11–30)	9 (7–13)	<0.001
30-day mortality, *n* (%)	25 (4.1)	20 (9.5)	5 (1)	<0.001
TnT I [ng/L]	615 (362–1111)	870 (443–1297)	499 (247–751)	<0.001
TnT II [ng/L]	675 (389–1458)	1081 (380–1782)	515 (218–812)	<0.001
**Main procedures**				
AVR, *n* (%)	313 (51)	55 (26)	258 (65)	0.11
AVP, *n* (%)	12 (2)	3 (1)	9 (22)	0.25
AVR + MVR, *n* (%)	53 (9)	34 (16)	19 (5)	0.91
AVR + MVP, *n* (%)	10 (2)	6 (2.8)	4 (1)	0.3
AVP + MVP, *n* (%)	2 (0.3)	0 (0)	2 (1)	0.74
MVP, *n* (%)	92 (15)	44 (21)	48 (15)	0.47
MVR, *n* (%)	107 (7)	63 (30)	44 (12)	0.17
TVR, *n* (%)	7 (1)	4 (2)	3 (1)	0.11
**Concomitant procedure**				
TVP, *n* (%)	164 (27)	106 (50)	58 (14)	0.01
CABG, *n* (%)	79 (15)	29 (17)	50 (13)	0.64

Data were characterized by median (Q1–Q3) and frequency (%). Abbreviations: AVP = aortic valve plasty, AVR = aortic valve replacement, CABG = coronary artery bypass graft, CRP = C-reactive protein, GFR = glomerular filtration rate, LV = left ventricle, MVP = mitral valve plasty, MVR = mitral valve replacement, NT-proBNP = N-terminal of the prohormone brain natriuretic peptide, TnT = preoperative troponin t, TnT I = troponin T measured in 0 day after surgery, TnT II = troponin T measured in the first day after surgery, TVP = tricuspid valve plasty, and TVR = tricuspid valve replacement.

**Table 2:** Analysis of predictive factors for the occurrence of primary endpoint.

	Univariate Analysis			Multivariate Analysis		
Variable	Odds Ratio	95% Cl	*p*-Value	Odds Ratio	95% Cl	*p*-Value
Age, years	1.035	1.018–1.052	<0.001	1.022	1.002–1.043	0.03
Female sex, *n* (%)	1.855	1.321–2.605	<0.001			
TnT, ng/L	1.911	1.521–2.402	<0.001			
NT-proBNP, pg/mL	1.950	1.659–2.292	<0.001	1.570	1.304–1.891	<0.001
CRP, mg/dL	1.610	1.337–1.938	<0.001			
EuroSCORE II, %	1.379	1.265–1.503	<0.001			
LV ejection fraction, %	0.961	0.946–0.976	<0.001			
Stroke in history, *n* (%)	2.100	1.015–4.344	0.04			
Atrial fibrillation, *n* (%)	5.386	3.746–7.742	<0.001	3.072	1.982–4.759	<0.001
Hemoglobin level, g/dL	0.763	0.684–0.851	<0.001			
GFR, mL/min/1.73 m^2^), *n* (%)	0.966	0.955–0.976	<0.001			
TnT I, ng/L	2.922	2.244–3.805	<0.001			
TnT II, ng/L	2.676	2.107–3.390	<0.001	1.933	1.492–2.506	<0.001
Rethoracotomy, *n* (%)	6.198	3.653–10.516	<0.001	5.518	2.800–10.876	<0.001

Abbreviations: CRP = C-reactive protein, LV = left ventricle, TnT = preoperative troponin t, TnT I = troponin T measured in 0 day after surgery, TnT II = troponin T measured in the first day after surgery, and NT-proBNP = N-terminal of the prohormone brain natriuretic peptide.

**Table 3:** Analysis of predictive factors for the occurrence of secondary endpoint.

	Univariate Analysis			Multivariate Analysis		
Variable	Odds Ratio	95% Cl	*p*-Value	Odds Ratio	95% Cl	*p*-Value
CRP, mg/dL	1.793	1.293–2.485	<0.001			
NT-proBNP, pg/mL	1.650	1.260–2.162	<0.001			
TnT, ng/L	2.062	1.433–2.967	<0.001			
EuroSCORE II, %	1.542	1.072–1.241	<0.001			
Hemoglobin level, g/dL	0.665	0.551–0.803	<0.001	0.696	0.558–0.87	0.001
Hs-TnT I, ng/L	3.388	2.312–4.966	<0.001			
Hs-TnT II, ng/L	3.440	2.370–4.992	<0.001	3.413	2.328–5.005	<0.001

Abbreviations: CRP = C-reactive protein, NT-proBNP = N-terminal of the prohormone brain natriuretic peptide, TnT = preoperative troponin t, TnT I = troponin T measured in 0 day after surgery, and TnT II = troponin T measured in the first day after surgery.

## Data Availability

The original contributions presented in this study are included in the article. Further inquiries can be directed to the corresponding author.
